# Air pollution burden of disease over highly populated states in the Middle East

**DOI:** 10.3389/fpubh.2022.1002707

**Published:** 2023-01-06

**Authors:** Rima J. Isaifan

**Affiliations:** Division of Sustainable Development (DSD), College of Science and Engineering, Hamad Bin Khalifa University (HBKU), Qatar Foundation (QF), Education City, Doha, Qatar

**Keywords:** air pollution, AirQ+, WHO, health impact, burden of disease, mortality, fine particulate matter, Middle East

## Abstract

**Background:**

Recent epidemiological research has proven that air pollution triggers the risk of morbidity and mortality due to respiratory and cardiovascular-related diseases. More specifically, fine particulate matter with a diameter of <2.5 μm (PM_2.5_) can penetrate deeply into the lung and bloodstream, causing critical adverse effects on human health.

**Objective:**

It is found that there is inadequate published research related to the health impact of ambient air pollution in the Middle East region. Some states are well studied, while others are not. This work aims to evaluate the health impact of long-term exposure to PM_2.5_ in the nine most populated countries in the Middle East region, with a total population of about 363 million (in 2012).

**Methods:**

In this study, the human health impacts in terms of total mortality and the estimated attributable proportion (AP) due to long-term exposure to ambient PM_2.5_ were estimated using the World Health Organization method and software (AirQ+).

**Results:**

In 2012, the annual median PM_2.5_ concentrations ranged from 34 μg/m^3^ in Turkey and Syria to 108 μg/m^3^ in Saudi Arabia. The total estimated mortalities in the nine most populated countries in the Middle East due to long-term exposure to fine particulate matter was about 152,925 (half of which were residents in Egypt). Moreover, the relative risk (RR) was the highest for Saudi Arabia at 1.8031 and the lowest for Turkey and Syria at a value of 1.1553. The highest AP (central value) was 44.5% in Saudi Arabia, while the lowest was 13.4% in Turkey and Syria.

**Conclusions:**

The results indicate a significant impact of air pollution due to long-term exposure to fine particles resulting in early mortality. This urges the collaboration between the governments and different sectors to adopt stringent regulations to control the anthropogenic sources related to traffic and industrial emissions in the Middle East in order to reduce the health burden of air pollution.

## 1. Introduction

Epidemiological investigations have shown that ambient air pollutants, exacerbated by climate change and other anthropogenic sources, increase the risk of respiratory, cardiovascular diseases, as well as lung cancer and would cause early mortality (1, 2). Air pollution is caused by several ambient harmful substances such as particulate matter (coarse and fine), nitrogen oxide, ozone, sulfur dioxide, and black carbon (3, 4). However, fine particulate matter with a diameter smaller than 2.5 μm (PM_2.5_) is of critical impact since it can penetrate into the lung's alveoli by inhalation causing adverse effects on human health (5, 6).

The ambient PM_2.5_ exposure caused about 1.1 million deaths due to ischemic heart diseases (IHD), and 240,000 deaths related to chronic obstructive pulmonary diseases (COPD) in 2012 worldwide (7). Particulate air pollution also directly impacts the material and human welfare such as buildings, historic monuments, paints, vehicles, and solar panels, among others (8, 9). Moreover, studies indicated a significant association between ambient PM_2.5_ inhalation and the incidence of several health endpoints such as asthma, bronchitis, and lung cancer (LC) related mortalities (10–12).

The Middle East region includes around 16 countries with a total population of 452.7 million in 2020. The long and short-term impact of air pollution on human health has not been well studied in all the countries in the regions. Several research papers have reported on selected adverse impacts on health in Iran (1, 13) and Saudi Arabia (14, 15), but the impact in other countries have not been reported, or are rarely studied (13, 16).

In order to facilitate the estimation of the long and short term health impact due to extensive exposure to air pollution, the World Health Organization (WHO) has developed an air quality health risk assessment impact model (AirQ+) (17, 18). This model is used to evaluate the burden of disease due to different air pollutants such as PM_2.5_, course particulate (PM_10_), nitrogen dioxide (NO_2_), ozone (O_3_), and black carbon (BC). The software has been used to report on several health endpoints around the world (5, 7, 19, 20).

The main objective of this study is to estimate the long-term impact of PM_2.5_ pollution exposure in the nine most populated countries in the Middle East (Egypt, Iran, Turkey, Iraq, Saudi Arabia, Yemen, Syria, United Arab Emirates (UAE), and Jordan) during 2012. The outcomes were evaluated in terms of total mortalities, attributable cases, and relative risk using AirQ+ model.

## 2. Materials and methods

### 2.1. Study area location

**Figure 1:**
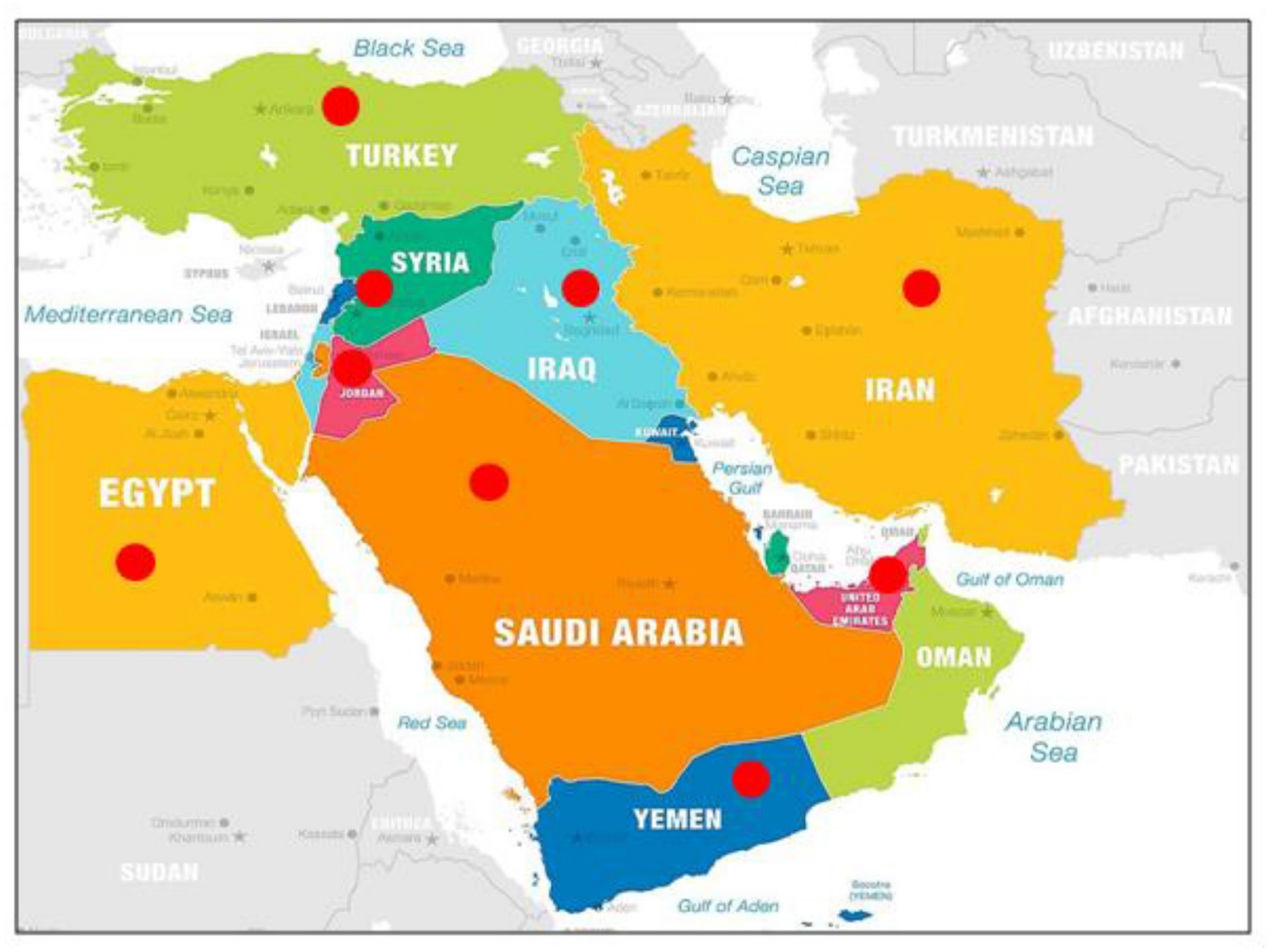
Map of the Middle East countries. Red circles indicate the states included in this study (21).

The Middle East (ME) is a geopolitical term that commonly refers to the region that includes around 16 countries with a total population of about 452.7 million in 2020. However, this study involves only the most populated countries with a total population equal or greater than 8 million in 2012 to match the year when PM_2.5_ levels were reported for all states. These include Egypt, Iran, Iraq, Turkey, Saudi Arabia, Yemen, Syria, United Arab Emirates (UAE), and Jordan which are all labeled with a red circle in Figure 1. The region is characterized by hot and dry climate in general with arid and semi-arid environment in most territories (22).

Table 1 lists the nine countries included in this study in order from the most populated to the least which are: Egypt, Iran, Turkey, Iraq, Saudi Arabia, Yemen, Syria, UAE, and Jordan. The total population in 2012 ranged from 82.3 million in Egypt to 8.1 in Jordan.

**Table 1:** The population in 2012 of the most populated nine countries in the Middle East selected in this study.

**Country**	**Population (million)**
Egypt	82.3
Iran	78.9
Turkey	74.9
Iraq	33.7
Saudi Arabia	28.7
Yemen	25.6
Syria	22.5
UAE	8.1
Jordan	8.1
Total	362.8

### 2.2. Air quality data

The annual PM_2.5_ concentrations for the selected countries were adopted from the World Health Organization report (Ambient air pollution: A global assessment of exposure and burden of disease) (7). This is due to the absence of consistent published measured PM_2.5_ concentrations for each country for the same duration. Table 2 shows the minimum, maximum and median average concentrations of fine particulate matter over the nine selected countries in 2012. It can be seen that the minimum annual PM_2.5_ concentration has exceeded the annual World Health Organization (WHO) 2006 Air Quality Guideline value of 10 μg/m^3^ for all the countries. This value has been reviewed and stated by the WHO in 2021 to be 5 μg/m^3^ which further indicates that there is almost no minimum PM_2.5_ concentration for safe exposure without inducing risk on the human health. For the long-term PM_2.5_ exposure health impact in terms of mortality evaluation, relative risk, and the attributable proportion (AP), the annual median PM_2.5_ concentrations were considered. The rational in this case is to avoid extremities when sudden short-term events take place such as the sandstorms which elevate PM concentration significantly. It is worth noting that some of the countries in this study witness such events, but not the others (23–25). Hence; excluding outlier values is preferred for the sake of outcome comparison on the same basis.

**Table 2:** The minimum, median, and maximum annual PM_2.5_ concentrations in rural and urban areas over the selected countries, adopted from WHO report (7).

	**PM**_**2.5**_ **(**μ**m/m**^**3**^**) rural and urban (2012)**		
**Country**	**Median**	**Min**	**Max**
Egypt	93	50	171
Iran	42	27	63
Turkey	34	22	50
Iraq	50	18	141
Saudi Arabia	108	67	174
Yemen	43	11	173
Syria	34	12	98
UAE	64	39	104
Jordan	36	24	53

### 2.3. Health impact assessment tool

In order to estimate the long-term impact of air pollution due to the exposure to fine particulate matter in the most populated countries in the Middle East, the Air Quality Health Impact Assessment (AirQ+) tool developed by the WHO was used. The software evaluates the mortality and morbidity in terms of several health endpoints such as respiratory diseases, daily hospitalization, lung cancer, COPD and IHD. It can be also used to evaluate the impact of several air pollutants other than PM_2.5_ which include coarse particulate matter, ozone, black carbon, and nitrogen oxides. The main outcome is the relative risk (RR) which is estimated *via* the following equation (1):


(1) $$\begin{array}{l} \text{RR}=\\ \frac{\text{Incidence in the exposed population to the air pollutant}}{\text{Incidence in the non exposed population to the air pollutant}} \end{array}$$

**Figure 2:**
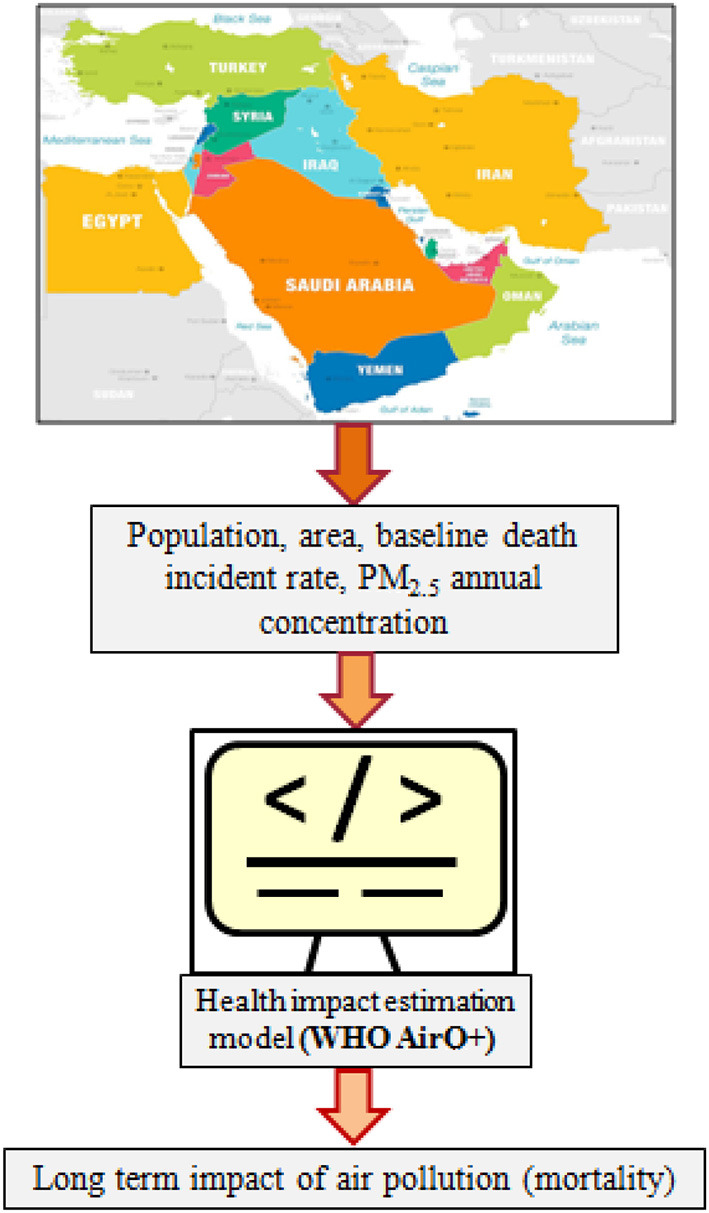
The steps followed in the methodology in this work.

Figure 2 shows the flow of the steps related to the methodology in this work to obtain the long-term impact of PM_2.5_ exposure. The input data for each country include the area in km^2^, the total population in the year of the study, the average annual concentration of the criteria pollutant (in this case PM_2.5_), and the base line incidence rate (in this case mortality). The output data shall include the relative risk of total mortality, the attributable total number of cases and percentage.

## 3. Results and discussion

It has been reported that there are several natural sources of air pollution in the Middle East region, such as windblown dust (26, 27), however, intensive anthropogenic activities related to urban development, industrial activities and transportation are the most dominant sources of air pollutants that played a critical role in increasing the air pollution burden of disease (23, 28–30).

**Figure 3:**
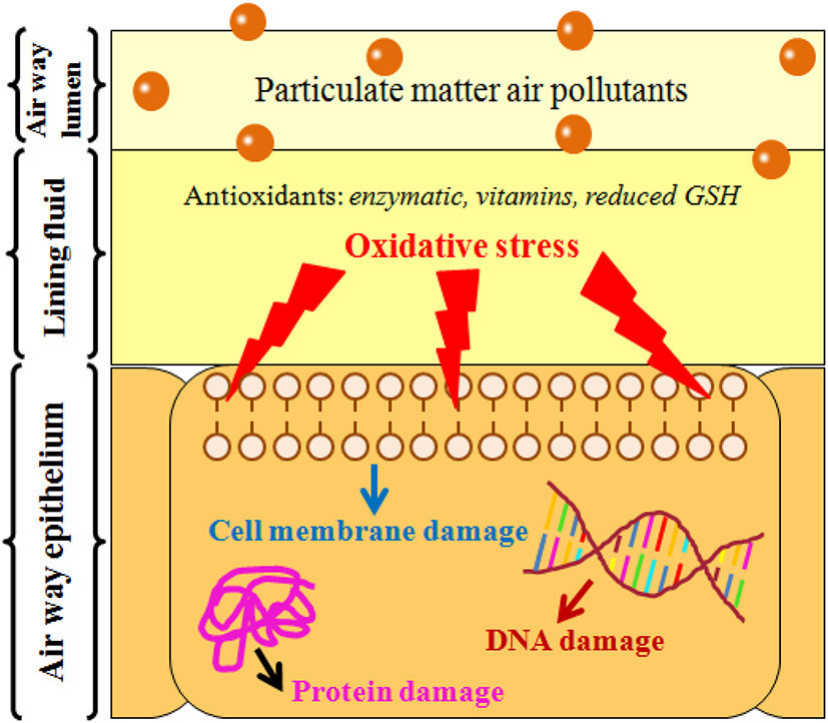
Mechanisms of airway injury by inhaling particulate matter.

Recent studies have reported on the adverse health impacts due to PM_2.5_ exposure (31–33). In order to better understand the impact of particulate matter on inducing several diseases in human beings, the mechanism shall be briefly described. At concentrations as low as 30 μg/m^3^, the epidemiological evidence suggests that PM_2.5_ that contains large number of soluble toxic metals are capable of redox cycling and oxidative stress (34). Oxidative stress refers to any disturbance in the pro- and anti-oxidant balance that would lead to a potential damage, and was first introduced in biology by Sies (35). More specifically, air pollutants contain various free radicals or highly oxidative gases. Upon inhaling the particulate matter, the release of reactive oxygen from lung cells attacks and oxidizes other cell components in the lungs. This leads to tissue, protein, and deoxyribonucleic acid (DNA) damage caused by the inflammatory reaction. The role of fine particulate matter to induce oxidative stress is represented schematically in Figure 3.

It can be shown that the oxidative stress plays a critical role to explain the mechanism of how air pollution adversely impacts human health when they are in contact with human cells and tissues (36). The generated reactive oxidative stress (ROS) overwhelms the antioxidant defenses in the body. Consequently, the ROS causes several pathological consequences due to the reactions that might take place with the DNA, lipids, and proteins in the cells. If these reactions were not stopped, they eventually cause a permanent damage of the cell that ends up with its death (36).

Table 3 shows the relative risk of mortality obtained from AirQ+ due to the long-term exposure to PM_2.5_. The relative risk indicates the probability of experiencing certain health effect (in this case, mortality) when certain pollutant (in this case, PM_2.5_) concentration increases at 10 μg/m^3^ increments (5).

**Table 3:** The relative risk (RR) of total mortality due to long term exposure to PM_2.5_ in the highest populated ME countries.

**Country**	**Lower**	**Central**	**Upper**
Egypt	1.3847	1.6475	1.9382
Iran	1.1337	1.2122	1.2906
Turkey	1.0987	1.1553	1.2109
Iraq	1.1698	1.2720	1.3756
Saudi Arabia	1.4686	1.8031	2.1845
Yemen	1.1381	1.2195	1.3009
Syria	1.0987	1.1553	1.2109
UAE	1.2310	1.3754	1.5259
Jordan	1.1073	1.1692	1.2303

The results show that the central values of relative risk ranges from 1.1553 for Turkey and Syria to 1.8031 for Saudi Arabia under the median PM_2.5_ concentration. That indicates that for each 10 μg/m^3^ increase in PM_2.5_ concentration, the risk for mortality increases by 80.31% for Saudi Arabia, and so on for other countries. In Iran, the relative risk for total mortality ranged between 1.1337 and 1.2906 over all the country. Similarly, Kermani et al., estimated the short-term effects of ambient air pollution in Iran. They found that PM_2.5_ had the most significant impact on the health of 19,048,000 residents in eight Iranian cities, causing total mortality of 5,670 out of 87,907 total deaths during a one-year time-period in 2012 (31). Borsi et al. (18), recently studied the health endpoints due to the exposure to several criteria air pollutants (ozone, PM_10_, SO_2_, and NO_2_) in Ahvaz city in Iran using AirQ+ model. They found that the annual cases of cardiovascular mortality due to PM_10_ exposure during the period 2010-2014 have reached up to 506 deaths yearly. Their results also showed that the number of hospital admissions due to cardiovascular disease related to ambient PM_10_ exposure was up to 586 during the same period. In another recently published work, Moradi et al. (37), estimated the health effects due to long and short term exposure to PM_2.5_ in Ardabil/ Iran using AirQ+ model. The first observation was that the PM_2.5_ average concentration in Ardabil in 2018 was around 15 μg/m^3^. This value is significantly less than the reported average values in 2012 by the World Health Organization as presented in Table 2 for Iran with an annual PM_2.5_ concentration range of 22–50 μg/m^3^. Moreover, the outcome of their research showed that exposure to ambient particulate matter, even at low concentrations, is associated with an increased risk of overall mortality and hospital admissions for respiratory and cardiovascular diseases. More specifically, a total number of deaths due to acute lower respiratory infections (ALRI), COPD, lung cancer, ischemic heart diseases, and stroke on average during the study period were estimated to be 73, 11, 7, 15, and 14; respectively (37).

It is worth noting that there is lack of research in this domain for several countries in the Middle East. In Rome, which is close to the ME region, the attributable proportion cases due to PM_2.5_ exposure was 16.5% for ischemic heart diseases and 8.5% for chronic obstructive pulmonary diseases mortality in 2015. Moreover, 947 premature deaths due to ischemic heart diseases and 244 due to chronic obstructive pulmonary diseases could have been avoided if PM_2.5_ concentrations would have not exceeded 10 μg/m^3^ (5). Moreover, Sicard et al. (20), reported that the relative risk for total mortality was about 1.062 and 1.015 in Iran and France; respectively.

The main sources of PM_2.5_ in the Middle East region have been attributed to anthropogenic sources which should be controlled and minimized (28, 30, 38). Momtazan et al. (39), drew attention to the adverse health impact due to air pollution and dust storms witnessed in the southwestern part of Iran. Their study investigated the relationship between hospital admissions for cardiovascular diseases and dust storms in Abadan and Khorramshahr (located southwest in Iran) in 2014–2016. They focused on estimating the number of people suffering from cardiovascular diseases attributed to PM_10_ exposure during the period 2014, 2015, and 2016, which were found to be 237, 259, and 274; respectively. They also reported that there was a significant relationship between PM_10_ concentrations in dusty days and the cases of cardiovascular diseases in both cities. In a 10-year data study conducted by Dastoorpoor et al. (40), to investigate the relation between air pollution and cardiovascular hospital admissions in Ahwaz/Iran, they found that there was a significant increase in hospital admissions and women's population specially in the case of ozone exposure. Moreover, there was a significant increase in hospital admissions for cardiovascular diseases in the whole population as well as gender and age groups associated with nitrogen dioxide (NO_2_) and nitrogen monoxide (NO) ambient levels.

A recent review by Nasser et al. (38), identified the major sources of air pollution in the urban cities in the main Middle East countries. In Egypt, the main air pollution sources attributed to human activities were due to traffic, industrial emissions, and burning in the open air. In Jordan, the emissions were mainly related to traffic. In Kuwait and Saudi Arabia, in addition to traffic, the emissions were attributed to industrial oil combustion and power plants emissions. In Lebanon and Turkey, the main sources were related to waste burning and traffic. In Qatar, the sources were attributed to traffic as well as to mega construction projects. In the UAE, air pollution was mainly due to industrial activities as well as traffic and marine activities in the ports. In Iran, emissions from anthropogenic sources were mainly due to transportation, industries and dust storms (41–43).

**Figure 4:**
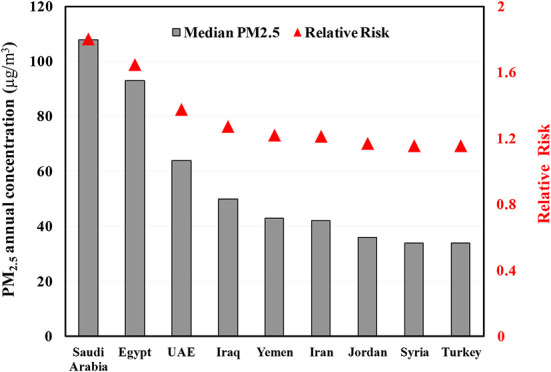
Relation between PM_2.5_ concentration and the relative risk of total mortality.

Moreover, the results show that the relative risk is significantly linked with the annual concentration of PM_2.5_ as shown in Figure 4.

The highest risk is corresponding with the highest PM_2.5_ concentration. This is due to the fact that the RR is proportional with the air pollutant concentration as per the following equation (7):


$$\begin{array}{l} RR=exp\left[\beta \left(x-x_{o}\right)\right] \end{array}$$

Where; *x* is the annual average mean concentration of the air pollutant (in this case PM_2.5_), *x*_*o*_ represents the background concentration of PM_2.5_ (10 μg/ m^3^) as per the World Health Organization air quality guidelines, and β is the risk function coefficient.

The results suggest that stringent measures should be taken to reduce the impact of air pollution in the urban context. Several studies suggested the use of clean transportation (30, 44), green vegetation planning of urban cities (45–47), and the use of nanotechnology to remove pollutants from the air (48–50). Among the most efficient feasible strategies to abate particulate pollution is the use of urban trees that require minimum irrigation and care needs and are well suited to grow in any location. These trees can be efficient to reduce particulate matter concentration in the air by acting as barriers for the incoming polluted air flow and by capturing air borne particles over the sticky or structured surface of the leaf (46). Other studies have shown the impact of using clean fuel and electric vehicles in urban cities to reduce the emissions of particulate matter (30). Moreover, the use of catalytic convertor filters embedded with noble nanomaterials has proven to remove around 95% of particulate matter emissions before they are released to the atmosphere (30).

Moreover, Table 4 shows that attributable percentage of total annual mortalities that are linked with long-term exposure to fine particulate ranges from 13.4% for Turkey and Syria to 39.3% for Egypt.

**Table 4:** Total mortality due to air pollution in the highest populated ME countries due to PM_2.5_ exposure.

**Country**	**Estimated AP (%)**			**Estimated total annual cases**			**Estimated cases per 100,000**	**Study period**	**References**
	**Lower**	**Central**	**Upper**	**Lower**	**Central**	**Upper**			
**Egypt**	27.8	39.3	48.4	52,870	74,785	92,109	133	2012	Current study
		–			43,531		–		(4)
**Turkey**	8.9	13.4	17.4	23,177	34,683	44,934	56	2012	Current study
		–			32,668		–		(4)
**Iran**	11.8	17.5	22.5	11,114	16,499	21,220	80	2012	Current study
		–			26,267		–		(4)
		6.45			5,670			(2011–2012)	(27)
**Iraq**	14.5	21.4	27.3	9,539	14,050	17,941	73	2012	Current study
		–			10,085		–		(4)
**Saudi Arabia**	31.9	44.5	54.2	5,861	8,181	9,960	71	2012	Current study
		–			8,119		–		(4)
**Yemen**	12.1	18.0	23.1	1,165	1,728	2,221	27	2012	Current study
		–			2,411		–		(4)
**Syria**	8.9	13.4	17.4	898	1,344	1,741	18	2012	Current study
		–			2,097		–		(4)
**Jordan**	9.7	14.5	18.7	693	1,034	1,338	27	2012	Current study
		–			1,483		–		(4)
**UAE**	18.8	27.3	34.5	428	621	784	14	2012	Current study
		–			655		–		(4)

**Figure 5:**
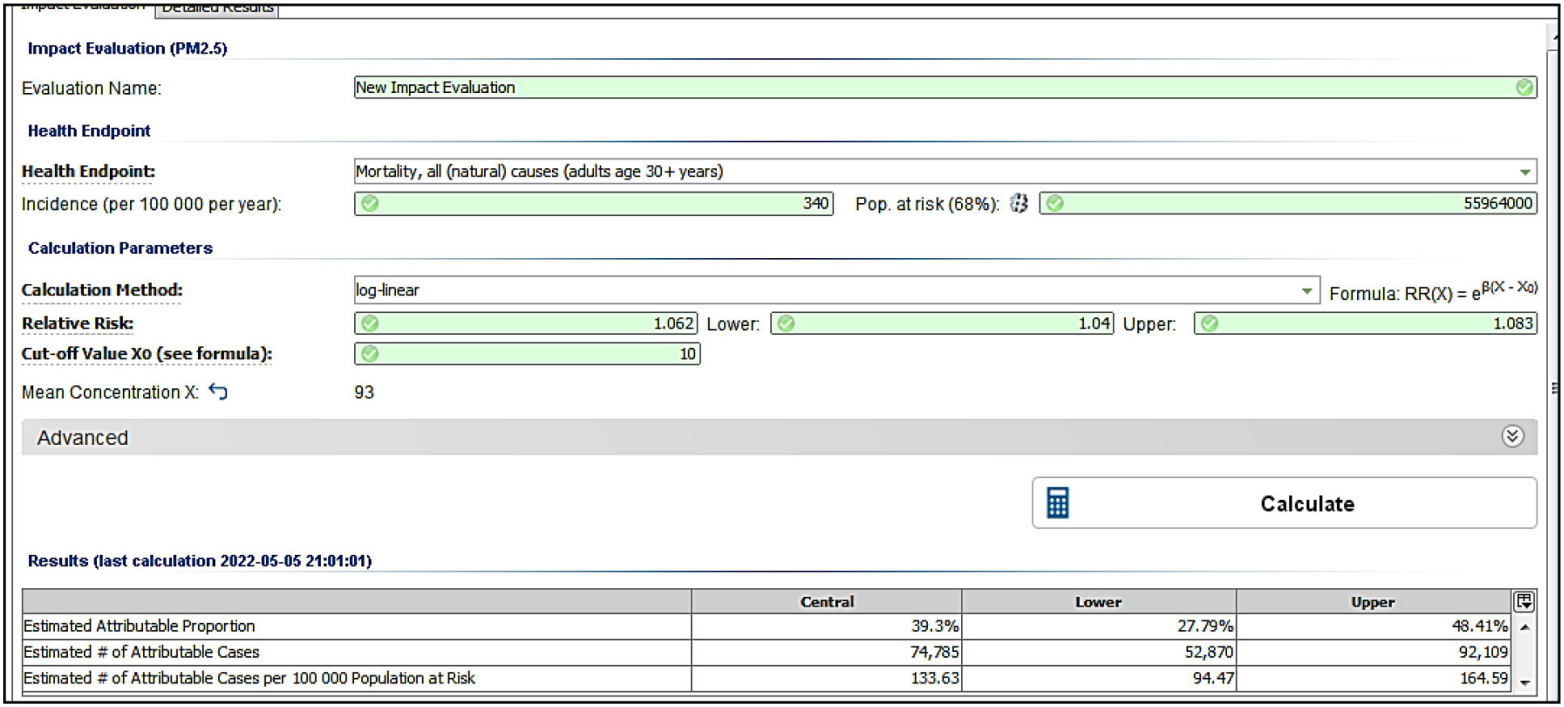
An example of the outcomes as per AirQ+ screen for Egypt.

The outcomes of AirQ+ software is also shown in Figure 5 for Egypt as an example. The results also indicate that for each 100,000 deaths in Egypt, 133 are attributed to diseases linked with exposure to PM_2.5_ air pollution.

The results in Table 4 were compared with some studies that do not cover the whole country, but rather one city or some cities per research cited (Table 4). For instance, Khaniabadi et al., (17) estimated that the relative risk for total mortality due to PM_2.5_ exposure in one city in Iran (Khorramabad, 540,000 inhabitants) was in the range of 1.015-1.019. In another study related to Dubai and Abu Dhabi in the UAE (51), it was estimated that 307,667 health care visits due to cardiovascular diseases were attributable to ambient PM_10_ daily exposure. The total overall estimated mortalities in this work have summed up to 152,925 compared with the World Health Organization report for the same year that summed up to 127,316 from the same 9 countries. The main difference was the lack of accurate demographic data for some countries by age group which caused the exposed population to vary. The lack of research that estimates the health impact of air pollution is mainly due to the lack of accurate measured or published air quality data on regular basis. This has also impacted the fact that there have been no national strategies aiming at assessing and mitigating the adverse health impact of air pollution (38).

## 4. Conclusions

In conclusion, the air pollution in the Middle East specially where high population with anthropogenic activities prevail have caused critical health issues that increased the burden of disease in the region. However, several countries with high population lack research on the impact of ambient air pollution on human health.

In order to estimate the long-term impact of PM_2.5_ exposure, the AirQ+ software was used to estimate the total mortality over the nine most populated countries in the Middle East. The results are in alignment with the published data by the World Health Organization for 2012. The highest annual mortalities were found in Egypt, while the lowest in the UAE.

The results of our study can be of substantial importance for researchers, policy makers, medical, public health, and scientific experts. Also, this study can influence the governments for the adoption of laws to minimize air pollution and achieve international air quality standards. The main recommendation is to urge local authorities to propose strict policies to reduce the emissions from traffic and other anthropogenic sources. This can be attained through proper sustainable urban planning, use of new technologies in vehicles, and the encouragement to sue public transportation and clean vehicles.

Moreover, this work draws attention for the need to study the impact of long and short-term exposure to pollution. The obvious lack of published studies in several states in the Middle East, while abundance is available for others such as Iran, urges the research community to investigate widely in this domain.

## Data availability statement

The original contributions presented in the study are included in the article/supplementary material, further inquiries can be directed to the corresponding author.

## Author contributions

RI is the sole author and contributor to this manuscript which includes conceptualization, structuring, and writing of the article.
